# A failure of sleep-dependent consolidation of visuoperceptual procedural learning in young adults with ADHD

**DOI:** 10.1038/s41398-022-02239-8

**Published:** 2022-12-02

**Authors:** Ranin Ballan, Simon J. Durrant, Robert Stickgold, Alexandra Morgan, Dara S. Manoach, Yafit Gabay

**Affiliations:** 1 Department of Special Education, University of Haifa, Haifa, Israel; 2 Edmond J. Safra Brain Research Center for the Study of Learning Disabilities, University of Haifa, Haifa, Israel; 3 Lincoln Sleep Research Centre and School of Psychology, University of Lincoln, Lincoln, UK; 4 Harvard Medical School, Boston, MA USA; 5 Department of Psychiatry, Massachusetts General Hospital, Boston, MA USA; 6 Athinoula A. Martinos Center for Biomedical Imaging, Charlestown, MA USA; 7 Department of Psychiatry, Beth Israel Deaconess Medical Center, Harvard Medical School, Boston, MA USA

**Keywords:** Human behaviour, Long-term memory

## Abstract

ADHD has been associated with cortico-striatal dysfunction that may lead to procedural memory abnormalities. Sleep plays a critical role in consolidating procedural memories, and sleep problems are an integral part of the psychopathology of ADHD. This raises the possibility that altered sleep processes characterizing those with ADHD could contribute to their skill-learning impairments. On this basis, the present study tested the hypothesis that young adults with ADHD have altered sleep-dependent procedural memory consolidation. Participants with ADHD and neurotypicals were trained on a visual discrimination task that has been shown to benefit from sleep. Half of the participants were tested after a 12-h break that included nocturnal sleep (sleep condition), whereas the other half were tested after a 12-h daytime break that did not include sleep (wakefulness condition) to assess the specific contribution of sleep to improvement in task performance. Despite having a similar degree of initial learning, participants with ADHD did not improve in the visual discrimination task following a sleep interval compared to neurotypicals, while they were on par with neurotypicals during the wakefulness condition. These findings represent the first demonstration of a failure in sleep-dependent consolidation of procedural learning in young adults with ADHD. Such a failure is likely to disrupt automatic control routines that are normally provided by the non-declarative memory system, thereby increasing the load on attentional resources of individuals with ADHD.

## Introduction

Attention-deficit/hyperactivity disorder (ADHD) is one of the most common neurodevelopmental disorders, and it affects a substantial part of the population, with wide-ranged manifestations in several life domains, influencing academic performance, job maintenance, and family and personal life [1, 2]. Core symptoms of ADHD include inattention, hyperactivity, and impulsivity. Despite decades of research, the neurocognitive basis of ADHD is unclear. Traditionally, ADHD has been associated with executive function deficits due to prefrontal network dysfunction (e.g., ref. [3]), but a growing body of evidence suggests that reward-related processes [4–6] and procedural learning [7–10] are likely to be affected as well.

Procedural learning refers to the acquisition of skills, stimulus–response associations, and rules acquired incrementally [11]. Unlike the acquisition of declarative knowledge, which lends itself to explicit conscious recollection [12] procedural memory is typically implicit, inaccessible to conscious recollection [13] and its acquisition and memory are demonstrated through task performance [14]. Although declarative and procedural memories can be distinguished [15] both memory systems interact during real-world learning [16–18]. Therefore, even well-known procedural learning tasks are likely to involve a mixture of declarative and procedural-based processes, yet the relative contribution of each memory system to task performance depends on the nature of the training experience [16, 19] as well as on the learning phase [20, 21]. The procedural memory system has been traditionally associated with the acquisition of motor skills [22], but a growing body of evidence implicates its involvement in the acquisition of perceptual [23], cognitive [24, 25], and language-related skills [26–29]. A procedural memory dysfunction could partially contribute to attentional deficits observed in ADHD, based on the notion that if simple procedural routines are not fully automatized, this can contribute to executive function deficits by overwhelming goal-directed behaviors [30, 31] or by increasing the load on attentional resources.

Indeed, several theoretical models posit procedural learning deficiencies in those with ADHD. According to the Procedural Deficit Hypothesis, ADHD is characterized by a selective disruption in the procedural memory structures that subserve the acquisition of skills, habits, and procedures, with declarative memory-based structures playing a compensatory role [32, 33]. Striatal memory dysfunction is presumed to give rise to impulsive behaviors that characterize those with ADHD [34] and is likely to affect implicit learning as well [35]. In addition, neurobiological models of ADHD posit that a deficit in non-declarative habit learning and memory is likely to arise from dopamine dysfunction within the neostriatum [36].

Consistent with these assumptions, functional and anatomical abnormalities are observed in core structures of the procedural memory system in individuals with ADHD [34, 37–39] who also possess lower striatal dopamine levels and have abnormally high densities of dopamine transporters [40, 41]. At the behavioral level, individuals with ADHD tend to be impaired in a variety of motor and cognitive procedural learning tasks, such as the finger tapping motor sequence task [42–45], probabilistic learning [7–9], artificial grammar learning [46], and visual category learning [10]. Yet the impairment may not affect all aspects of performance [47] and depends on the nature of the learning task [46]. For example, in the study conducted by Barnes and his colleagues [47], inconsistent progression of sequence learning was observed in children with ADHD compared to control children, although the overall learning score was intact.

Nonetheless, there is no complete understanding of procedural memory functions in those with ADHD. Many studies examined procedural learning in ADHD in a single training session [7–10, 46, 47], thus disregarding important processes involved in skill acquisition. Procedural skill learning is a multi-stage, dynamic process that entails gradual performance changes across time. In addition to performance gains that occur concurrently with repeated exposure to a given task, delayed performance gains may also evolve in the absence of additional practice [48]. These latter changes involve consolidation processes, whereby formed memory traces become less susceptible to interference, and are transformed and honed to represent new knowledge [48, 49]. Ample evidence suggests that post-training sleep can benefit the consolidation of newly learned skills into long-term memory across visual [50, 51], motor [52], cognitive [53, 54], and language-related [55, 56] domains. Although both the stabilization and off-line improvement of motor skills may evolve over time spent awake [57, 58], the post-training enhancement of various forms of procedural memories (e.g., visual procedural learning) seems to be primarily dependent on sleep [50, 51, 59–61]. The predominant assumption is that sleep-dependent memory consolidation may function by increasing the efficiency of memory retrieval and activation through brain plasticity [62]. Consistently, accumulating evidence suggests that REM sleep, especially after spindle-rich stage 2 sleep, is important for the improvement of procedural memories [50, 59, 60, 63]. This is especially relevant with regard to neurodevelopmental disorders such as ADHD in which sleep problems are considered to be an integral part of its presentation [64–67] and in which altered sleep architecture is observed [68–73]. These observations raise the possibility that the altered sleep mechanisms that characterize those with ADHD [69, 74–76] could contribute to their procedural learning deficits, similar to deficits observed in other learning domains [77–80].

Although difficulties in the consolidation of procedural memories have been previously implicated in ADHD [42, 44, 81–83], it is still impossible to conclude whether the observed deficits are specifically related to sleep. In previous studies, consolidation was measured after post-training sleep [42, 44, 81, 82], but the lack of a wakefulness condition and overnight sleep polysomnography measures make it difficult to associate these findings with a specific sleep-dependent mechanism. Furthermore, in studies in which wake vs. sleep conditions or sleep measures were included, the role of sleep in consolidating procedural memories in ADHD was examined by employing the Serial Reaction Time Task [83, 84]. Although this type of task is well-suited for studying the consolidation of procedural memories in general [57, 85, 86], it may not be optimal for examining sleep-dependent consolidation processing, as mounting evidence suggests that performance (implicit sequence learning) is not enhanced by sleep [57, 87–95]. Performance on explicit motor sequence learning tasks, on the other hand, is facilitated by sleep [52, 58]. Indeed, in the study of Prehn–Kristensen, Molzow [83] typical children did not exhibit sleep-dependent offline gains in the SRT task, making it difficult to draw conclusions about the role sleep plays in mediating procedural memory consolidation of this task in individuals with ADHD. Just as important, although sleep problems in individuals with ADHD continue from childhood to adulthood [96, 97], we are aware of no studies that examined the influence of sleep on procedural memory consolidation of adults with ADHD.

In this study, we aimed to fill this gap by using a visual discrimination task (VDT) to assess sleep-dependent consolidation of procedural memory in young adults with ADHD. The VDT requires rapid discrimination of the orientation of a target embedded in distractors. In such tasks, a target screen is presented briefly and is followed, after a variable interstimulus interval (ISI), by a mask screen, and performance is usually measured as the minimum ISI at which performance remains above 80% accuracy. The VDT is a well-known task for assessing skill learning [50, 51, 98–100], which is defined as experience-dependent improvements in performance in perceptual, perceptuomotor, or motor tasks [101]. The knowledge gained in the VDT is hard to describe verbally and can be communicated through task performance. Consistently, amnestic patients with medial temporal lobe damage who are unable to acquire declarative knowledge [102], exhibit intact learning and overnight consolidation when tested on the VDT, despite having no conscious recollection of taking the test previously [23]. Therefore, the VDT task appears to be a procedural learning task with no role for the medial temporal lobe in the learning and consolidation of the task. In neurotypicals, improvement on the VDT develops slowly after training [50, 103], with no improvement when retesting occurs on the same day as training. Instead, improvement is observed exclusively overnight and is associated with the presence of initial slow wave sleep (SWS) and late rapid eye movement (REM) sleep [50, 51, 61]. In this study, participants with ADHD and neurotypicals completed the VDT task using wakefulness vs. sleep design to determine whether alterations in consolidating procedural memories in adults with ADHD can be specifically attributed to sleep. If people with ADHD are characterized by a selective (sleep-dependent) failure in procedural memory consolidation, they are not expected to show the same offline improvement in the VDT following a sleep interval, but rather to be on par with neurotypicals during a wakefulness period.

## Methods

### Participants

Sixty-seven university students (26 with ADHD and 41 controls) took part in the study. All participants had no history of neurological disorders and/or psychiatric disorders and had normal or corrected-to-normal vision and normal hearing. Previous studies used a similar sample size when examining memory consolidation in ADHD populations [42, 77, 79, 81, 83]. The inclusion criteria for the ADHD group included (1) a formal diagnosis of ADHD by a licensed clinician, (2) positive screening for ADHD based on the adult ADHD self-report scale (ASRS), namely a score ≥ 51, (3) lack of a formal diagnosis of a comorbid developmental disorder such as developmental dyslexia, and (4) an IQ estimate within the normal range (Raven score >10th percentile). The control group was comprised of individuals with no history of learning disabilities who exhibited no difficulties in attentional skills (e.g., did not receive a positive score for ADHD based on the ASRS) and were at the same level of cognitive skills (assessed by the Raven test) as the ADHD group. Based on this criterion, one ADHD participant was removed from the final sample. The data of one control participant removed from the final sample due to an experimentation error. Finally, participants from both groups were not included in the study if they reported sleep-related disorders as measured by the Pittsburgh Sleep Quality Index; (PSQI [104]) A cutoff score of ≥6 has been used to maintain high sensitivity of the test and maximize diagnostic accuracy among college students [105]. The Institutional Review Board of the University of Haifa approved the study, which was conducted in accordance with the Declaration of Helsinki, with written informed consent provided by all participants. Participants received a compensation of NIS 120 (approximately $37) for participating in the study.

Participants completed the Raven’s SPM tests as an estimate of their cognitive abilities, as well as the ASRS questionnaire. Participants also completed measures of sleep quality (Pittsburgh Sleep Quality; PSQI, [104]) and alertness (Stanford Sleepiness Scale; SSS, [106]) prior to the VDT task. Details of the tests are presented in Table 1 and results are shown in Table 2. The groups did not differ significantly in age or nonverbal IQ estimates. Naturally, the ADHD group differed significantly from the control group on the ADHD measures derived from the ASRS questionnaire.

**Table 1:** List of psychometric tests.

Ability	Test	Description
**INTELLECTUAL ABILITY**	**Raven’s Standard Progressive Matrices test** (Raven, Court, & Raven, [123]).	Raven’s Standard Progressive Matrices test—Nonverbal intelligence was assessed by the Raven’s SPM test. This task requires participants to choose an item from the bottom of the figure that completes the pattern at the top. The maximum raw score is 60. Test reliability coefficient is 0.9.
**INATTENTION AND A HYPRACTIVITY/IMPULSIVITY SCALE**	**Adult ADHD Self-Report Scale (ASRS)** (Zohar & Konfortes, [124])	The ADHD Self-Report Scale (ASRS; Zohar & Konfortes, [124]) is an 18-item self-report scale that relates directly to the DSM IV TR diagnostic criteria. Part A of the scale consists of 6 items and part B of the remaining 12 items. Participants mark an X in the appropriate boxes. ASRS questions ask respondents how often a specific symptom of ADHD has occurred to them over the past six months on a five-point response scale ranging from 0 to 4 (0 = never, 1 = rarely, 2 = sometimes, 3 = often, and 4 = very often). Total scores range from 0 to 72. The Hebrew version of the ASRS-v1.1 was developed by the World Health Organization (WHO) as the official Hebrew version.
**SLEEP QUALITY**	**Pittsburgh Sleep Quality Index (PSQI)** (Buysse et al. [104]).	The PSQI questionnaire is a questionnaire that assesses sleep habits during the past month only. This questionnaire consists of Nineteen individual items that generate seven “component” scores: subjective sleep quality, sleep latency, sleep duration, habitual sleep efficiency, sleep disturbances, use of sleeping medication, and daytime dysfunction. The order of the PSQI items has been modified from the original order in order to fit the first 9 items (which are the only items that contribute to the total score) on a single page. Item 10, which is the second page of the scale, does not contribute to the PSQI score. In scoring the PSQI, seven component scores are derived, each scored 0 (no difficulty) to 3 (severe difficulty). The component scores are summed to produce a global score (range 0 to 21). Higher scores indicate worse sleep quality.
**SLEEPINESS SELF-ASSESSMENT**	**Stanford Sleepiness Scale (SSS)** (Hoddes et al. [106]).	The Stanford Sleepiness Scale is a self-assessment questionnaire, developed by Hoddes et al. [106]. Participants fill out the Stanford Sleepiness Scale to measure alertness, just prior to the specific task training. At the Stanford Sleepiness Scale participants will be reported bedtime, wake time, how they feel at the given moment (that is to rate the degree of sleepiness) in the range of 1 = Feeling active, vital, alert, or wide awake to 7 = No longer fighting sleep, sleep onset soon; having dream-like thoughts. With higher scores indicating sleepier and the lowest scores indicating more activity.

**Table 2:** Demographic and psychometric data of the ADHD and control groups.

Measurement	Control group M (SD)	ADHD group M (SD)	*t* value	*P*
Age (in years)	24.68 (2.57)	24.48 (3.01)	0.29	ns
Intellectual ability				
Raven test	54.14 (4.47)	53.44 (4.69)	0.60	ns
Attentional functions				
ASRS	32.63 (6.67)	66.52 (8.28)	−18.1	<0.01
Sleep Quality Index				
PSQI	3.07 (1.23)	4.12 (1.3)	−3.27	<0.05
Sleep self-assessment				
Stanford Sleepiness Scale (SSS) Session 1	1.90 (0.73)	1.84 (0.55)	0.36	ns
Stanford Sleepiness Scale (SSS) Session 2	2.02 (0.68)	2.20 (0.91)	−0.88	ns

### Visual discrimination task

The VDT task [59] was adapted from the study of Stickgold et al. [51]. Participants performed the task in a dark room sitting in front of a computer screen while placing their head on a chin rest 35–40 cm from the computer screen. Before beginning the actual experiment, participants practiced the task with the experimenter (Introduction phase). The experimenter explained to the participants what they were going to see in each trial using PowerPoint slides and made sure that the participants understood what they were required to do. Then, the participants practiced the task with the experimenter until they were able to reach eight correct answers in a row. Immediately after the introduction phase, the participants performed the VDT task.

**Fig. 1:**
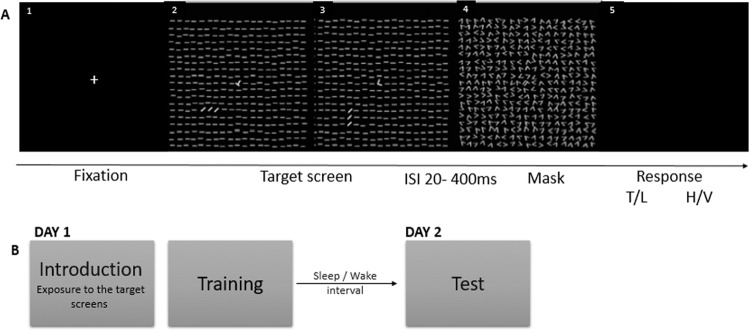
Visual Discrimination Task. Task procedure (**A**) At first, a fixation screen appeared (**A1**). After participants pressed on the keyboard a target screen was presented for 16 ms (**A2**, **A3**) that contained a rotated “T” or “L” at the center and a horizontal or vertical array of three diagonal bars in the lower-left quadrant. A blank screen followed the target screen for an ISI of 20–400 ms and then a mask for 16 ms (**A4**). Upon termination of the mask screen, the screen went black (**A5**) and participants indicated with button presses whether there had been a T or an L at fixation and whether the diagonal lines were arranged horizontally (H) or vertically (V). This was followed by the return of the fixation screen. **B** Timeline of the experiment: the first session was divided into two parts: an introduction phase and a training phase. After a 12-h sleep/wake interval, participants performed a test phase.

At the beginning of each trial of the VDT task, participants were presented with a black screen with a white cross in the center. Participants were instructed to fixate their gaze on the cross during each trial throughout the experiment. When ready, participants pressed the spacebar and were presented with a target screen (Fig. 1A) for 17 msec, followed by a blank screen. After an interstimulus interval (ISI) of 20–400 ms, the blank screen was replaced with a mask screen (Fig. 1A). After the presentation of the mask screen, participants were required to indicate whether the central letter on the target screen had been a “T” or an “L” and whether the three diagonal bars that appeared in the lower-left quadrant were arranged horizontally or vertically.

Each block was comprised of 50 trials with a constant ISI. Both training and test phases were composed of one block each at ISIs of 400, 300, 200, and 160 ms, followed by three blocks each at ISIs of 120 down to 20 ms, in decrements of 20 ms, presented in order of decreasing ISI. The task was discontinued if a participant was not able to correctly identify the arrangement of the diagonal lines in at least 66% of the trials for two consecutive blocks. Therefore, the length of each training session was determined based on the participants’ performance and varied across participants from 30 to 35 min in the first session and 15 to 20 min in the second session. Following the approach of Stickgold, Whidbee [50] the outcome measure was the detection threshold defined as the interpolated ISI at which the participant’s accuracy for reporting the arrangement of the diagonal lines dropped to 80%.

#### Procedure

Participants performed the VDT task using a sleep vs. wake design and were randomly assigned to sleep vs. wake groups. Participants in the sleep groups were trained on the VDT task in the evening and tested after a 12-h interval that included sleep (evening [7–9 p.m.] to morning [7–9 a.m.]), whereas the wake groups were trained in the morning and tested after a 12-h interval that did not include sleep (morning [7–9 a.m.] to evening [7–9 p.m.]) (see Fig. 1B). At the beginning of each session, participants filled out the Stanford Sleepiness Scale (SSS; Hoddes et al. [106]) to measure alertness. Matlab with Psychtoolbox controlled stimulus presentation and recording of response time and accuracy.

#### Approach to analyses

Performance was assessed by averaging the detection thresholds defined as the interpolated ISI at which the participant’s accuracy for reporting the arrangement of the diagonal lines dropped to 80%. To examine group differences in initial learning a factorial ANOVA was conducted, with Group (Controls vs. ADHD) and Condition (Sleep vs. Wake) as between-subject factors, with mean threshold as the dependent variable during the training session. To assess offline gains a mixed ANOVA was conducted, with Group (Controls vs. ADHD) and Condition (Sleep vs. Wake) as between-subject factors and Session (Training vs. Test) as the within-subject factor, with mean threshold as the dependent variable. An additional ANOVA was conducted by using proportional threshold improvement [(Test-Training)/Training] as the dependent variable. The Kolmogorov–Smirnov and the Levine tests were used to determine whether the distributions obeyed the assumptions of normality and homogeneity, respectively. Some of the variables had distributions that departed from normality and homogeneity assumptions. ANOVA is considered a robust test against violations of assumptions. Yet, theoretically meaningful comparisons that were found to be significant were also analyzed with nonparametric tests (Mann–Whitney *U* tests). In addition, for the proportional improvement analysis, which is the critical measure for testing consolidation effects in terms of what is preserved relative to initial learning, all assumptions were met. A correlational analysis was conducted to determine whether there is an association between sleep-dependent memory consolidation on the VDT task and sleep quality assessed the Pittsburgh Sleep Quality Index within each of the sleep groups separately.

## Results

### Initial learning

**Fig. 2:**
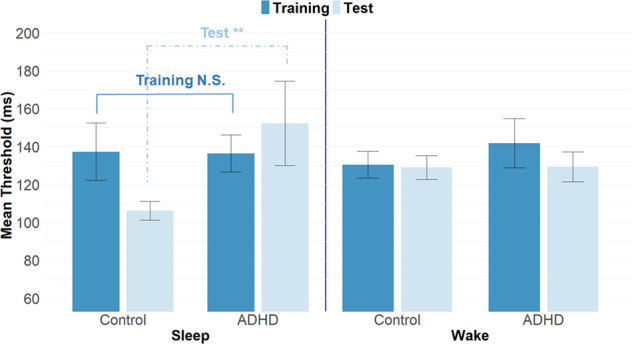
Mean detection threshold in the VDT task as a function of group, session, and condition. Error bars represent one standard error.

Results are presented in Fig. 2. None of the main effects or interaction were significant (all *F’s* < 1, all *P’s* > 0.66).

### Offline gains

Results are presented in Fig. 2. The only significant effect was the triple interaction of Group × Session × Condition, *F*(1, 61) = 5.8, *P* = 0.01, *η*_*p*_² = 0.08. To understand the basis for this interaction, we compared consolidation between the ADHD vs. control groups using 2 (Group) × 2 (Session) ANOVAs conducted for each condition (sleep vs. wake) separately. In the sleep condition there was a significant Group × Session interaction, *F*(1, 61) = 8.22, *P* = 0.005. Further analysis revealed that the control group exhibited a significant improvement in average detection threshold during sleep, *F*(1, 23) = 2.48, *P* = 0.02 from training (M_training_ = 137.26, S.E._training_ = 15.16) to test (M_Test_ = 106.20, S.E._Test_ = 4.97), whereas such an improvement was absent in the ADHD group, *t*(13) = 0.14, *P* = 0.88 (M_training_ = 136.31, S.E._training_ = 9.78, M_Test_ = 152.27, S.E._Test_ = 22.26). Thus, an interval of sleep produced significant learning benefits in neurotypicals, consistent with previous findings of overnight sleep-dependent enhancement of VDT performance, but not in ADHD participants. For the wakefulness condition, the Group × Session interaction was not significant, *F*(1, 61) = 0.38, *P* = 0.53. In addition to the ANOVA analyses, a nonparametric Mann–Whitney *U* test was conducted to examine the differences between the two sleep groups on the test phase to address the possibility that variables departed from the assumptions of normality and homogeneity. This analysis revealed similar results to those of the ANOVA. In particular, the performance of the ADHD sleep group at the test phase was significantly inferior to that of the control sleep group (Mann–Whitney *U* test, one-tailed*, Z* = −2.12, *P* = 0.015).

### Proportional improvement

Analyses revealed a significant Group × Condition interaction, *F*(1,61) = 6.94, *P* = 0.01; *η*_*p*_^*2*^ = 0.10. Further analysis revealed that in the sleep condition, the control group exhibited a significant proportional improvement compared to the ADHD group, *F*(1,61) = 9.23, *P* = 0.003, while no group differences were observed in the wakefulness condition, *F* < 1.

### Correlations between sleep-dependent memory consolidation and sleep quality

No correlation was observed either in the control, *r*(23) = −0.312, *P* = 0.146., or in the ADHD sleep groups, *r*(13) = −0.243, *P* = 0.424.

## Discussion

In this study, we tested the role of sleep in consolidating skill memory in adults with ADHD using a visual discrimination task that has been shown to benefit from sleep. Consistent with previous investigations [50, 51], we observed that neurotypical participants exhibited delayed (offline) gains in task performance of the VDT following a sleep interval, which are considered a behavioral expression of consolidation processes. These offline gains were absent among participants who remained awake in the interval between sessions. Contrary to observations in neurotypicals, as a group, young adult participants with ADHD failed to exhibit sleep-dependent offline gains. Interestingly, in the wakefulness condition and the pre-sleep session of the sleep condition, participants with ADHD were on a par with neurotypicals with regard to speed of visual perception.

Taken together, these findings suggest the first demonstration of a failure of sleep-dependent consolidation of procedural learning in young adults with ADHD. This raises several hypotheses as to the locus of the observed behavioral deficit. First, it could be the case that factors unrelated to memory consolidation (such as visuospatial processing or processing speed) produced the behavioral deficit we observed. For example, the VDT may impose demands on processing speed that could influence the performance of special populations. We judge this possibility as less likely based on two observations. First, in the present study participants with ADHD exhibited a similar level of performance as the neurotypical group during the first session. Factors unrelated to memory consolidation should be manifested in online learning and not selectively influence offline processes. Second, we observed that the wake group of ADHD participants was on par with neurotypicals with regard to their visual discrimination skills both at training and during the test. It is unclear why factors unrelated to memory consolidation would influence behavioral performance in the sleep condition but not the wakefulness condition. This difference may arise due to cohort differences; however, the fact that the performance of ADHD participants in the first session was similar in the sleep vs. wake conditions reduces the possibility that factors unrelated to memory consolidation were driving the group differences we observed. As noted above, even well-known procedural learning tasks are likely to involve a mixture of declarative and procedural processes. However, despite the possibility that initial skill acquisition might involve declarative components in which learners are likely to verbally rehearse information required for the execution of a skill [20, 107], later learning phases involve gradual improvement in performance without relying on verbal mediation [107]. The fact that group differences were observed during consolidation rather than initial learning supports the assumption of a procedural memory deficit in people with ADHD. Furthermore, amnestic patients are capable of demonstrating intact learning when trained on the VDT [23]. This also lessens the possibility that declarative-based processes are likely to be involved during initial learning phases of the VDT. A second possibility for the selective (sleep-dependent) memory consolidation deficit we observed in ADHD might be related to the possibility that sleep quality was reduced in our ADHD sample, which could give rise to the sleep-dependent behavioral deficit observed. A third possibility is that altered sleep architecture contributed to the behavioral deficit we observed in those with ADHD. In this regard, no correlation was observed between sleep quality (assessed by the Pittsburgh Sleep Quality Index) and sleep-dependent improvement on the VDT, in either of the ADHD/control sleep groups. This reduces the possibility that moderating variables such as sleep quality could drive the group differences. Notably, unless sleep quality is very disrupted (which was unlikely in our sample, in which only ADHD and controls below the cutoff score in the Pittsburgh Sleep Quality Index were included), it is not expected to correlate with sleep-dependent improvement on the VDT. On the other hand, sleep oscillations (a continuum of brain activity across sleep–wake states) are likely to correlate with sleep-dependent improvement [50, 51, 59, 108]. Our behavioral data indicate that sleep-dependent consolidation of procedural learning is disrupted in those with ADHD, however, future studies are needed to specify the exact sleep-mediated mechanism by which such a disruption occurs.

Procedural memory consolidation in the VDT task has been associated with the presence of initial slow wave sleep (SWS) and late rapid eye movement (REM) sleep stages [50, 51, 61]. Other studies demonstrated that a nap could be as effective as sleep for inducing memory consolidation processes in the VDT [60]. Mednick, Nakayama [60] showed that if REM was missing from the nap, sleep-dependent memory consolidation did not occur, therefore highlighting the role of brain activity during this specific sleep stage in consolidating visual procedural memories. Interestingly, while there are reports of diverse abnormalities of sleep macro- and micro-architecture in ADHD [69, 74–76], REM sleep alterations are one of the most consistent findings [76] but see also [73, 109–111]. Several studies revealed a reduced percentage of REM sleep [96, 112] and a reduced REM latency in ADHD [75, 113, 114] yet see also [115], though the clinical significance of REM sleep alterations in ADHD remains unknown [96]. Therefore, alteration in brain activity occurring during this specific phase of sleep could contribute to the diminished consolidation of skill memories observed here in ADHD, as more REM sleep has previously been associated with improved procedural memory consolidation [116].

While the present behavioral findings provide initial support for the hypothesis that sleep-dependent consolidation of procedural learning is disrupted in ADHD, the exact altered mechanism is difficult to specify and will have to be addressed in future studies using polysomnography measures. This study nevertheless suggests that previous investigations examining procedural learning in ADHD in one training session [46] missed the deficit in sleep-dependent procedural memory consolidation. It also raises the possibility that atypical consolidation of procedural memories observed previously in ADHD [42, 44, 81] is specific to sleep. Our results are in particular resonant with previous research reporting impaired sleep-dependent memory consolidation in ADHD in other domains [77] and extend it into the realm of procedural memory.

Our findings have important implications for understanding the neurocognitive basis of ADHD. In particular, post-training sleep is important for the development of skill automaticity [117] and signature indicators of the establishment of robust and efficient long-term “how to” memory representations [49, 118–121]. If simple sleep-dependent routines do not fully automatize procedural learning in ADHD, this can contribute to executive function deficits by overwhelming goal-directed behaviors or by exhausting attentional resources. Thus, disrupted sleep-dependent procedural memory consolidation processes can partially give rise to the attentional deficits that characterize those with ADHD. This assumption is consistent with theoretical models positing procedural memory abnormalities in ADHD [32, 34, 36].

Consolidation of procedural memories occurs in the context of normal sleep architecture [62] and it will therefore be important for future studies to determine whether the behavioral deficit observed in this study is associated with specific sleep mechanisms in ADHD. Here we observed that memory consolidation that normally induces changes in attentional processing (the speed of identification of targets among distractors) is impaired in ADHD. Future studies are nevertheless needed to determine whether the sleep-associated pattern observed in the present study generalizes to other forms of skill memories. Finally, future studies should examine the influence of pharmacological manipulations on the sleep-dependent procedural memory consolidation of people with ADHD. Evidence suggests that such manipulations aid sleep-dependent procedural memory consolidation of patients who suffer from basal ganglia dysfunction [122], which could be tested in ADHD populations as well.

To conclude, the present study examined the role of sleep in supporting the consolidation of procedural memories in ADHD. We present the first demonstration that sleep does not produce skill memory benefits in ADHD as it does for neurotypicals. This pattern of results occurred in the context of intact initial learning and was specific to sleep. We suggest that difficulties in fully automating simple routines during normal sleep are likely to overwhelm goal-directed behaviors or to exhaust attentional resources in ADHD, thus partially contributing to their attentional deficits.

## References

[1] Stern A, Pollak Y, Bonne O, Malik E, Maeir A (2017). The relationship between executive functions and quality of life in adults with ADHD. J Atten Disord.

[2] Wehmeier PM, Schacht A, Barkley RA (2010). Social and emotional impairment in children and adolescents with ADHD and the impact on quality of life. J Adolesc Health.

[3] Barkley RA (1997). Behavioral inhibition, sustained attention, and executive functions: constructing a unifying theory of ADHD. Psychological Bull.

[4] Luman M, Oosterlaan J, Sergeant JA (2005). The impact of reinforcement contingencies on AD/HD: a review and theoretical appraisal. Clin Psychol Rev.

[5] Sagvolden T, Aase H, Zeiner P, Berger D (1998). Altered reinforcement mechanisms in attention-deficit/hyperactivity disorder. Behav Brain Res.

[6] Scheres A, Milham MP, Knutson B, Castellanos FX (2007). Ventral striatal hyporesponsiveness during reward anticipation in attention-deficit/hyperactivity disorder. Biol Psychiatry.

[7] Gabay Y, Shahbari-Khateb E, Mendelsohn A (2018). Feedback Timing modulates probabilistic learning in adults with ADHD. Sci Rep..

[8] Gabay Y, Goldfarb L (2017). Feedback-based probabilistic category learning is selectively impaired in attention/hyperactivity deficit disorder. Neurobiol Learn Mem.

[9] Frank MJ, Santamaria A, O’Reilly RC, Willcutt E (2007). Testing computational models of dopamine and noradrenaline dysfunction in attention deficit/hyperactivity disorder. Neuropsychopharmacology..

[10] Huang-Pollock CL, Maddox WT, Tam H (2014). Rule-based and information-integration perceptual category learning in children with attention-deficit/hyperactivity disorder. Neuropsychology..

[11] Knowlton BJ, Siegel AL, Moody TD. Procedural learning in humans. 2017.

[12] Eichenbaum H (1997). How does the brain organize memories?. Science..

[13] Ashby FG, Crossley MJ (2012). Automaticity and multiple memory systems. Wiley Interdiscip Rev: Cogn Sci.

[14] Koziol LF, Budding DE. Procedural learning. Encycl Sci. Learn. 2012:2694–6.

[15] Squire LR (1992). Declarative and nondeclarative memory: multiple brain systems supporting learning and memory. J Cogn Neurosci.

[16] Packard MG, Goodman J (2013). Factors that influence the relative use of multiple memory systems. Hippocampus..

[17] Crossley MJ, Ashby FG (2015). Procedural learning during declarative control. J Exp Psychol: Learn Mem Cognition.

[18] Squire LR, Dede AJ (2015). Conscious and unconscious memory systems. Cold Spring Harb Perspect Biol.

[19] Gabay Y (2021). Delaying feedback compensates for impaired reinforcement learning in developmental dyslexia. Neurobiol Learn Mem.

[20] Anderson JR (1982). Acquisition of cognitive skill. Psychological Rev.

[21] Newell A, Rosenbloom P. Mechanisms of skill acquisition. Cognitive skills and their acquisition. 1981.

[22] Doyon J, Penhune V, Ungerleider LG (2003). Distinct contribution of the cortico-striatal and cortico-cerebellar systems to motor skill learning. Neuropsychologia..

[23] Stickgold R. Human studies of sleep and off-line memory reprocessing. Oxford: Oxford UP; 2003. p. 42–63.

[24] Knowlton BJ, Mangels JA, Squire LR (1996). A neostriatal habit learning system in humans. Science..

[25] Shohamy D, Myers C, Onlaor S, Gluck M (2004). Role of the basal ganglia in category learning: how do patients with Parkinson’s disease learn?. Behav Neurosci.

[26] Durrant SJ, Cairney SA, Lewis PA (2013). Overnight consolidation aids the transfer of statistical knowledge from the medial temporal lobe to the striatum. Cereb Cortex.

[27] Ullman MT (2004). Contributions of memory circuits to language: the declarative/procedural model. Cognition..

[28] Lim S-J, Fiez JA, Holt LL (2019). Role of the striatum in incidental learning of sound categories. Proc Natl Acad Sci USA.

[29] Feng G, Yi HG, Chandrasekaran B (2019). The role of the human auditory corticostriatal network in speech learning. Cereb Cortex.

[30] Redgrave P, Rodriguez M, Smith Y, Rodriguez-Oroz MC, Lehericy S, Bergman H (2010). Goal-directed and habitual control in the basal ganglia: implications for Parkinson’s disease. Nat Rev Neurosci.

[31] Manoach DS (2003). Prefrontal cortex dysfunction during working memory performance in schizophrenia: reconciling discrepant findings. Schizophrenia Res.

[32] Ullman MT, Pullman MY (2015). A compensatory role for declarative memory in neurodevelopmental disorders. Neurosci Biobehav Rev.

[33] Nicolson RI, Fawcett AJ (2007). Procedural learning difficulties: reuniting the developmental disorders?. TRENDS Neurosci.

[34] Goodman J, Marsh R, Peterson BS, Packard MG (2014). Annual research review: the neurobehavioral development of multiple memory systems–implications for childhood and adolescent psychiatric disorders. J Child Psychol Psychiatry.

[35] Nigg JT, Casey B (2005). An integrative theory of attention-deficit/hyperactivity disorder based on the cognitive and affective neurosciences. Dev Psychopathol.

[36] Sagvolden T, Johansen EB, Aase H, Russell VA (2005). A dynamic developmental theory of attention-deficit/hyperactivity disorder (ADHD) predominantly hyperactive/impulsive and combined subtypes. Behav Brain Sci.

[37] Sobel LJ, Bansal R, Maia TV, Sanchez J, Mazzone L, Durkin K (2010). Basal ganglia surface morphology and the effects of stimulant medications in youth with attention deficit hyperactivity disorder. Am J Psychiatry.

[38] Qiu A, Crocetti D, Adler M, Mahone EM, Denckla MB, Miller MI (2009). Basal ganglia volume and shape in children with attention deficit hyperactivity disorder. Am J Psychiatry.

[39] Cubillo A, Halari R, Smith A, Taylor E, Rubia K (2012). A review of fronto-striatal and fronto-cortical brain abnormalities in children and adults with attention deficit hyperactivity disorder (ADHD) and new evidence for dysfunction in adults with ADHD during motivation and attention. Cortex..

[40] Dougherty DD, Bonab AA, Spencer TJ, Rauch SL, Madras BK, Fischman AJ (1999). Dopamine transporter density in patients with attention deficit hyperactivity disorder. Lancet.

[41] Krause K-H, Dresel SH, Krause J, Kung HF, Tatsch K (2000). Increased striatal dopamine transporter in adult patients with attention deficit hyperactivity disorder: effects of methylphenidate as measured by single photon emission computed tomography. Neurosci Lett.

[42] Adi-Japha E, Fox O, Karni A (2011). Atypical acquisition and atypical expression of memory consolidation gains in a motor skill in young female adults with ADHD. Res Developmental Disabilities.

[43] Fox O, Adi-Japha E, Karni A. Motor memory consolidation processes in young female adults with ADHD may be less susceptible to interference. Neurosci Lett. 2017;637:91–5. 10.1016/j.neulet.2016.11.044 27888044

[44] Fox O, Karni A, Adi-Japha E (2016). The consolidation of a motor skill in young adults with ADHD: shorter practice can be better. Res Dev Disabilities.

[45] Fox O, Adi-Japha E, Karni A (2014). The effect of a skipped dose (placebo) of methylphenidate on the learning and retention of a motor skill in adolescents with attention deficit hyperactivity disorder. Eur Neuropsychopharmacol.

[46] Laasonen M, Väre J, Oksanen-Hennah H, Leppämäki S, Tani P, Harno H (2014). Project dyadd: implicit learning in adult dyslexia and ADHD. Ann Dyslexia.

[47] Barnes KA, Howard JH, Howard DV, Kenealy L, Vaidya CJ (2010). Two forms of implicit learning in childhood ADHD. Developmental Neuropsychol.

[48] Stickgold R, Walker MP (2005). Sleep and memory: the ongoing debate. Sleep..

[49] Dudai Y, Karni A, Born J (2015). The consolidation and transformation of memory. Neuron..

[50] Stickgold R, Whidbee D, Schirmer B, Patel V, Hobson JA (2000). Visual discrimination task improvement: a multi-step process occurring during sleep. J Cogn Neurosci.

[51] Stickgold R, James L, Hobson JA (2000). Visual discrimination learning requires sleep after training. Nat Neurosci.

[52] Walker MP, Brakefield T, Morgan A, Hobson JA, Stickgold R (2002). Practice with sleep makes perfect: sleep-dependent motor skill learning. Neuron..

[53] Pozzobon A, Fang Z, Al-Kuwatli J, Toor B, Ray L, Fogel S. Sleep enhances consolidation of memory traces for complex problem-solving skills. Cerebral Cortex. 2021;32:653–67. 10.1093/cercor/bhab216 34383034

[54] Djonlagic I, Rosenfeld A, Shohamy D, Myers C, Gluck M, Stickgold R (2009). Sleep enhances category learning. Learn Mem.

[55] Durrant SJ, Taylor C, Cairney S, Lewis PA (2011). Sleep-dependent consolidation of statistical learning. Neuropsychologia..

[56] Tamminen J, Payne JD, Stickgold R, Wamsley EJ, Gaskell MG (2010). Sleep spindle activity is associated with the integration of new memories and existing knowledge. J Neurosci.

[57] Simor P, Zavecz Z, Horváth K, Éltető N, Török C, Pesthy O (2019). Deconstructing procedural memory: different learning trajectories and consolidation of sequence and statistical learning. Front Psychol.

[58] Robertson EM, Pascual-Leone A, Press DZ (2004). Awareness modifies the skill-learning benefits of sleep. Curr Biol.

[59] Karni A, Tanne D, Rubenstein BS, Askenasy JJ, Sagi D (1994). Dependence on REM sleep of overnight improvement of a perceptual skill. Science..

[60] Mednick S, Nakayama K, Stickgold R (2003). Sleep-dependent learning: a nap is as good as a night. Nat Neurosci.

[61] McDevitt EA, Duggan KA, Mednick SC (2015). REM sleep rescues learning from interference. Neurobiol Learn Mem.

[62] Stickgold R, Walker MP (2007). Sleep-dependent memory consolidation and reconsolidation. Sleep Med.

[63] Strauss M, Griffon L, Van Beers P, Elbaz M, Bouziotis J, Sauvet F, et al. Order matters: sleep spindles contribute to memory consolidation only when followed by rapid-eye-movement sleep. Sleep. 2022;45:zsac022. 10.1093/sleep/zsac022 35037060

[64] Cohen-Zion M, Ancoli-Israel S (2004). Sleep in children with attention-deficit hyperactivity disorder (ADHD): a review of naturalistic and stimulant intervention studies. Sleep Med Rev.

[65] Brown TE, McMullen WJ (2001). Attention deficit disorders and sleep/arousal disturbance. Ann N. Y Acad Sci.

[66] Stephens RJ, Chung SA, Jovanovic D, Guerra R, Stephens B, Sandor P (2013). Relationship between polysomnographic sleep architecture and behavior in medication-free children with TS, ADHD, TS and ADHD, and controls. J Developmental Behav Pediatrics.

[67] Nigg JT. Getting ahead of ADHD: what next-generation science says about treatments that work? And how you can make them work for your child. Guilford Press; 2017.

[68] Kirov R, Kinkelbur J, Banaschewski T, Rothenberger A (2007). Sleep patterns in children with attention‐deficit/hyperactivity disorder, tic disorder, and comorbidity. J Child Psychol Psychiatry.

[69] Kirov R, Banaschewski T, Uebel H, Kinkelbur J, Rothenberger A (2007). REM-sleep alterations in children with co-existence of tic disorders and attention-deficit/hyperactivity disorder: impact of hypermotor symptoms. Eur Child Adolesc Psychiatry.

[70] Goraya JS, Cruz M, Valencia I, Kaleyias J, Khurana DS, Hardison HH (2009). Sleep study abnormalities in children with attention deficit hyperactivity disorder. Pediatr Neurol.

[71] Scarpelli S, Gorgoni M, D’Atri A, Reda F, De, Gennaro L (2019). Advances in understanding the relationship between sleep and attention deficit-hyperactivity disorder (ADHD). J Clin Med.

[72] Nigg JT (2013). Attention-deficit/hyperactivity disorder and adverse health outcomes. Clin Psychol Rev.

[73] Luongo A, Lukowski A, Protho T, Van Vorce H, Pisani L, Edgin J. Sleep’s role in memory consolidation: what can we learn from atypical development? Adv Child Dev Behav. 2021;60:229–60. 10.1016/bs.acdb.2020.08.001 33641795

[74] Konofal E, Lecendreux M, Cortese S (2010). Sleep and ADHD. Sleep Med.

[75] Kirov R, Kinkelbur J, Heipke S, Kostanecka‐Endress T, Westhoff M, Cohrs S (2004). Is there a specific polysomnographic sleep pattern in children with attention deficit/hyperactivity disorder?. J Sleep Res.

[76] Kirov R, Brand S (2014). Sleep problems and their effect in ADHD. Expert Rev Neurotherapeutics.

[77] Prehn-Kristensen A, Göder R, Fischer J, Wilhelm I, Seeck-Hirschner M, Aldenhoff J (2011). Reduced sleep-associated consolidation of declarative memory in attention-deficit/hyperactivity disorder. Sleep Med.

[78] Prehn-Kristensen A, Munz M, Molzow I, Wilhelm I, Wiesner CD, Baving L (2013). Sleep promotes consolidation of emotional memory in healthy children but not in children with attention-deficit hyperactivity disorder. PLoS ONE.

[79] Prehn-Kristensen A, Molzow I, Förster A, Siebenhühner N, Gesch M, Wiesner CD (2017). Memory consolidation of socially relevant stimuli during sleep in healthy children and children with attention-deficit/hyperactivity disorder and oppositional defiant disorder: what you can see in their eyes. Biol Psychol.

[80] Prehn-Kristensen A, Munz M, Göder R, Wilhelm I, Korr K, Vahl W (2014). Transcranial oscillatory direct current stimulation during sleep improves declarative memory consolidation in children with attention-deficit/hyperactivity disorder to a level comparable to healthy controls. Brain Stimulation.

[81] Fox O, Adi-Japha E, Karni A (2017). Motor memory consolidation processes in young female adults with ADHD may be less susceptible to interference. Neurosci Lett.

[82] Korman M, Levy I, Karni A (2017). Procedural memory consolidation in attention-deficit/hyperactivity disorder is promoted by scheduling of practice to evening hours. Front Psychiatry.

[83] Prehn-Kristensen A, Molzow I, Munz M, Wilhelm I, Müller K, Freytag D (2011). Sleep restores daytime deficits in procedural memory in children with attention-deficit/hyperactivity disorder. Res Dev Disabilities.

[84] Prehn-Kristensen A, Ngo H-VV, Lentfer L, Berghäuser J, Brandes L, Schulze L (2020). Acoustic closed-loop stimulation during sleep improves consolidation of reward-related memory information in healthy children but not in children with attention-deficit hyperactivity disorder. Sleep..

[85] Kóbor A, Janacsek K, Takács Á, Nemeth D (2017). Statistical learning leads to persistent memory: evidence for one-year consolidation. Sci Rep..

[86] Juhasz D, Nemeth D, Janacsek K (2019). Is there more room to improve? The lifespan trajectory of procedural learning and its relationship to the between-and within-group differences in average response times. PLoS ONE.

[87] Gabay Y, Schiff R, Vakil E (2012). Attentional requirements during acquisition and consolidation of a skill in normal readers and developmental dyslexics. Neuropsychology..

[88] Gabay Y, Schiff R, Vakil E (2012). Dissociation between online and offline learning in developmental dyslexia. J Clin Exp Neuropsychol.

[89] Meier B, Cock J (2014). Offline consolidation in implicit sequence learning. Cortex..

[90] Pan SC, Rickard TC (2015). Sleep and motor learning: is there room for consolidation?. Psychological Bull.

[91] Song S, Howard JH, Howard DV (2007). Sleep does not benefit probabilistic motor sequence learning. J Neurosci.

[92] Nemeth D, Janacsek K, Londe Z, Ullman MT, Howard DV, Howard JH (2010). Sleep has no critical role in implicit motor sequence learning in young and old adults. Exp Brain Res.

[93] Viczko J, Sergeeva V, Ray LB, Owen AM, Fogel SM (2018). Does sleep facilitate the consolidation of allocentric or egocentric representations of implicitly learned visual-motor sequence learning?. Learn Mem.

[94] Spencer RM, Sunm M, Ivry RB (2006). Sleep-dependent consolidation of contextual learning. Curr Biol.

[95] Conte F, Ficca G (2013). Caveats on psychological models of sleep and memory: a compass in an overgrown scenario. Sleep Med Rev.

[96] Sobanski E, Schredl M, Kettler N, Alm B (2008). Sleep in adults with attention deficit hyperactivity disorder (ADHD) before and during treatment with methylphenidate: a controlled polysomnographic study. Sleep..

[97] Mahajan N, Hong N, Wigal TL, Gehricke J-G (2010). Hyperactive-impulsive symptoms associated with self-reported sleep quality in nonmedicated adults with ADHD. J Atten Disord.

[98] Censor N, Sagi D, Cohen LG (2012). Common mechanisms of human perceptual and motor learning. Nat Rev Neurosci.

[99] Karni A, Sagi D (1991). Where practice makes perfect in texture discrimination: evidence for primary visual cortex plasticity. Proc Natl Acad Sci USA.

[100] Chambers AM, Payne JD. The memory function of sleep: How the sleeping brain promotes learning. In: Duarte A, Barense M, Addis RD (Eds.). The Wiley Blackwell handbook on the cognitive neuroscience of memory. West Sussex, UK: Wiley-Blackwell; 2015. pp. 218–43.

[101] Squire LR (1998). Memory systems. Comptes Rendus de l’Académie des Sci-Ser III-Sci de la Vie.

[102] Schacter DL, Wagner AD, Buckner RL. Memory systems of 1999. In: Tulving E, Craik FIM (Eds.). Oxford handbook of memory, Oxford University Press, New York; 2000. pp. 627–43.

[103] Karni A, Sagi D (1993). The time course of learning a visual skill. Nature..

[104] Buysse DJ, Reynolds CF, Monk TH, Berman SR, Kupfer DJ (1989). The Pittsburgh Sleep Quality Index: a new instrument for psychiatric practice and research. Psychiatry Res.

[105] Dietch JR, Taylor DJ, Sethi K, Kelly K, Bramoweth AD, Roane BM (2016). Psychometric evaluation of the PSQI in US college students. J Clin Sleep Med.

[106] Hoddes E, Zarcone V, Smythe H, Phillips R, Dement WC (1973). Quantification of sleepiness: a new approach. Psychophysiology..

[107] Fitts PM. Perceptual-motor skill learning. In: Categories of human learning. Academic Press, Elsevier; 1964. p. 243–85.

[108] Gais S, Plihal W, Wagner U, Born J (2000). Early sleep triggers memory for early visual discrimination skills. Nat Neurosci.

[109] Platon MR, Bueno AV, Sierra JE, Kales S (1990). Hypnopolygraphic alterations in attention deficit disorder (ADD) children. Int J Neurosci.

[110] Ringli M, Souissi S, Kurth S, Brandeis D, Jenni OG, Huber R (2013). Topography of sleep slow wave activity in children with attention-deficit/hyperactivity disorder. Cortex..

[111] Furrer M, Jaramillo V, Volk C, Ringli M, Aellen R, Wehrle FM (2019). Sleep EEG slow-wave activity in medicated and unmedicated children and adolescents with attention-deficit/hyperactivity disorder. Transl Psychiatry.

[112] Gruber R, Xi T, Frenette S, Robert M, Vannasinh P, Carrier J (2009). Sleep disturbances in prepubertal children with attention deficit hyperactivity disorder: a home polysomnography study. Sleep..

[113] O’Brien LM, Ivanenko A, Crabtree VM, Holbrook CR, Bruner JL, Klaus CJ (2003). Sleep disturbances in children with attention deficit hyperactivity disorder. Pediatr Res.

[114] Crabtree VM, Ivanenko A, Gozal D (2003). Clinical and parental assessment of sleep in children with attention-deficit/hyperactivity disorder referred to a pediatric sleep medicine center. Clin Pediatrics.

[115] Díaz-Román A, Mitchell R, Cortese S (2018). Sleep in adults with ADHD: systematic review and meta-analysis of subjective and objective studies. Neurosci Biobehav Rev.

[116] Rasch B, Born J. About sleep’s role in memory. Physiol Rev. 2013;93:681–766. 10.1152/physrev.00032.2012 PMC3768102 23589831

[117] Atienza M, Cantero JL, Stickgold R (2004). Posttraining sleep enhances automaticity in perceptual discrimination. J Cogn Neurosci.

[118] Dorfberger S, Adi-Japha E, Karni A (2007). Reduced susceptibility to interference in the consolidation of motor memory before adolescence. PLoS ONE.

[119] Karni A. The acquisition of perceptual and motor skills: a memory system in the adult human cortex. Cogn Brain Res. 1996. pp. 39–48 10.1016/s0926-6410(96)00039-0 9049069

[120] Karni A, Bertini G (1997). Learning perceptual skills: behavioral probes into adult cortical plasticity. Curr Opin Neurobiol.

[121] Seitz AR, Dinse HR (2007). A common framework for perceptual learning. Curr Opin Neurobiol.

[122] Grogan JP, Tsivos D, Smith L, Knight BE, Bogacz R, Whone A (2017). Effects of dopamine on reinforcement learning and consolidation in Parkinson’s disease. eLife..

[123] Raven JC, Court JH, Raven J. Manual for Raven’s Progressive Matrices and Vocabulary Scales. Oxford: Oxford Psychologists Press.1992.

[124] Zohar AH, Konfortes H (2010). Diagnosing ADHD in Israeli adults: the psychometric properties of the adult ADHD Self Report Scale (ASRS) in Hebrew. Isr J Psychiatry Relat Sci.

